# Remifentanil enhances the sedative effect of remimazolam during anesthesia induction in patients undergoing hysteroscopy: a randomized controlled trial

**DOI:** 10.1080/07853890.2025.2534850

**Published:** 2025-07-23

**Authors:** Zhengui Xie, Yeqing Liao, Qiuling Chen, Cuiwen Zhang, Jiaxin Luo, Huiyu Cao, Yuliu Lin, Hongmeng Lan, Xiaofang Huang, Xuehai Guan

**Affiliations:** Department of Anesthesiology, the First Affiliated Hospital of Guangxi Medical University, Nanning, Guangxi, PR China

**Keywords:** Anesthesia induction, hysteroscopy, remifentanil, remimazolam, sedation

## Abstract

**Background:**

Remimazolam is a novel, short-acting benzodiazepine. This study aimed to investigate whether remifentanil enhances the sedative effect of remimazolam during anesthesia induction in patients undergoing hysteroscopy.

**Methods:**

We included 258 adult patients who underwent hysteroscopy. Patients were randomly allocated to three groups (*n* = 86) in a 1:1:1 ratio. Patients in the saline group received no remifentanil but normal saline during anesthesia induction. Patients in the remifentanil-1 group received low-dose remifentanil (plasma concentration 1 ng/ml), and those in the remifentanil-2 group received higher doses of remifentanil (plasma concentration 2 ng/ml) during anesthesia induction. After reaching the preset plasma concentration of remifentanil, remimazolam was administered (5 mg/kg/h) until loss of consciousness (LOC). During anesthesia maintenance, remimazolam and remifentanil were changed to ensure that the MOAA/S score reached 0. The primary outcome was the time from remimazolam administration to LOC. The secondary outcomes included the doses of remimazolam until LOC, characteristics of the operation and anesthesia, vital signs, and adverse events.

**Results:**

The time from remimazolam administration to LOC was 154 (139, 180), 120 (104, 139), and 117 (105, 135) s in the saline group, remifentanil-1group, and remifentanil-2 groups, respectively (*p* < 0.05). The doses of remimazolam until LOC were 12(11, 13), 9.6(8.7, 11), and 8.9(7.9, 10) mg in the saline group, remifentanil-1group, and remifentanil-2 groups, respectively (*p* < 0.05). There was no significant difference in the characteristic of surgery and anesthesia between groups.

**Conclusion:**

Remifentanil enhances the sedative effect of remimazolam during anesthesia induction in patients undergoing hysteroscopy.

**Clinical trial registration:**

The trial was registered at http://www.chictr.org.cn (ChiCTR2200062983).

## KEY MESSAGES

- Remimazolam is a novel, short-acting benzodiazepine. Combinations of sedatives, including propofol and benzodiazepines, with opioids are commonly used for anesthesia.
- Remifentanil enhances the sedative effect of remimazolam during anesthesia induction in patients undergoing hysteroscopy.
- Combinations of opioids, such as remifentanil, with benzodiazepines, such as remimazolam, enhance the sedative effect of remimazolam during anesthesia induction.

## Introduction

Hysteroscopy is regarded as the gold standard for evaluating intracavity pathology. Its shift toward outpatient settings due to the adoption of ‘see and treat’ protocols, allowing for both diagnosis and treatment within the same session, reducing costs and improving patient convenience [1]. However, despite these advancements, many patients arrive with heightened pain expectations influenced by online discussions and social media [2]. This phenomenon often amplifies patient discomfort and may necessitate transitioning minimally invasive hysteroscopy to the operating room. Within this context, there is a critical need to adopt anesthetic protocols that balance patient comfort with safety, ensuring a minimally invasive approach.

Benzodiazepines (such as diazepam and midazolam) have been considered as adjuvants to general anesthesia since 1982. However, drawbacks such as individual variations in induction doses, long-acting residual effects, and slow onset render these drugs unsuitable for routine use in anesthesia induction or maintenance [3]. Remimazolam is a novel ultra-short-acting benzodiazepine sedative that acts on gamma-aminobutyric acid type A receptors (GABAARs). Remimazolam is rapidly metabolized into an inactive metabolite by tissue carboxylesterases. This resulted in a fast sedation offset. This sedative has recently been developed for endoscopic procedures [4,5] and general anesthesia [6–8] with the following characteristics of ideal anesthetic and sedative drugs: rapid onset and rapid offset [9,10], lower incidence of respiratory and circulatory depression [5–7,11], and no injection pain. Meanwhile, remimazolam is safely, effectively used for anesthesia and sedation in pediatric patients, cardiac surgery patients, and high-risk cardiovascular patients undergoing non-cardiac surgery [12–14]. In addition, the sedative effect of remimazolam can be reversed by flumazenil [15]. Rapid recovery can be achieved using remimazolam [16]. The development of remimazolam offers an opportunity to revisit benzodiazepine-based anesthesia [17–19].

Remifentanil is a novel ultrashort-acting opioid analgesic. Remifentanil has a strong analgesic effect and limited sedative effects [20]. Remifentanil has a characteristic of rapid onset and offset, and no accumulation with continuous infusion. Thus, remifentanil can achieve predictable recovery [21].

Combinations of sedatives, including propofol and benzodiazepines, with opioids are commonly used for anesthesia and procedural sedation [22–25]. Drug-drug interactions may change the pharmacological effects. The co-administration of drugs may improve efficacy but may also exaggerate adverse effects. Therefore, it is important to understand the characteristics of these interactions. To the best of our knowledge, few studies have focused on the interaction between remimazolam and remifentanil. The present study was designed to test the hypothesis that remifentanil enhances the sedative effect of remimazolam during anesthesia induction in patients undergoing hysteroscopy.

## Materials and methods

### Study design

This single-center, single-blinded, randomized, placebo-controlled clinical trial was conducted at the First Affiliated Hospital of Guangxi Medical University, between Aug 26, 2022 and Dec 31, 2023. The protocol was approved by the Medical Ethics Committee of the First Affiliated Hospital of Guangxi Medical University (2022-KY-E-266, Chairperson: Prof. Songqing He, approval date: Aug 5, 2022). The trial was registered prior to patient enrollment at chictr.org.cn (ChiCTR2200062983, Principal investigator: Xuehai Guan, Date of registration: Aug 26, 2022), adhered to the 2010 CONSORT statement, and was performed in accordance with the Declaration of Helsinki. All patients gave written informed consent prior to enrollment.

Two hundred and sixty-four patients who were scheduled for hysteroscopy by the surgeon in charge under general anesthesia (aged 18–60 years, ASA physical status I–II) were enrolled. Patients who meet any of the following criteria were excluded: difficult airway, severe hypertension (systolic blood pressure ≥180 mmHg, or diastolic blood pressure ≥110 mmHg), severe heart disease (NYHA class III or IV), severe liver dysfunction (Child–Pugh class B or C), chronic obstructive pulmonary disease, chronic kidney disease (glomerular filtration rate <60 ml min^−1^), severe anemia (hemoglobin level <60 g L^−1^), shock, hyperthyroidism, hypothyroidism, or gout caused by abnormal purine metabolism, electrolyte disorders, alcohol or drug dependency, delayed gastric retention or emptying, or allergy to remimazolam or remifentanil.

### Randomization and masking

Subjects were randomly allocated to three groups (normal saline group, remifentanil-1 group, and remifentanil-2 group) in a ratio of 1:1:1 using random numbers generated by EpiCalc 2000 software. Sealed opaque envelopes were used to conceal allocations. Envelopes were opened to prepare drugs by an assistor who was not involved in anesthesia or data collection. The allocation and received intervention were blinded to the patients, surgeons, and data collectors until data collection and analysis were completed.

### Anesthesia and interventions

All subjects fasted for 8 h, and only clear liquids were allowed for up to 2 h before surgery. The patient did not receive any premedication. After the patient entered the surgical waiting area, the nurse assisted in establishing a venous pathway, and Ringer’s lactate solution was started to maintain patency (4 ml kg^−1^. h^−1^). Routine monitoring was conducted as previously [26]. In brief, on arrival to the operating room, the patients were monitored for pulse oximetry saturation (SpO_2_), electrocardiography, non-invasive blood pressure (NIBP), bispectral index (BIS, Covidien, USA), and capnography. Prior to anesthesia induction, 100% oxygen was administered at a flow rate of 6 l min ^−1^ for 3 min by placing a mask on the face. During anesthesia induction, patients in the saline group received no remifentanil but normal saline, whereas those in the remifentanil-1 group received low-dose remifentanil (plasma concentration 1 ng. ml^−1^; Yichang Humanwell Pharmaceutical Co., China), and patients in the remifentanil-2 group received a higher dose of remifentanil (plasma concentration 2 ng ml^−1^). Remifentanil was administered by using a target-controlled infusion pump (Veryark, China). After remifentanil reached the preset plasma concentration, remimazolam tosilate (Jiangsu Hengrui, China; diluted with normal saline to 1 mg ml^−1^)) was administered at a rate of 5 mg kg^−1^ h^−1^ until loss of consciousness (LOC; modified observatory/sedation scales, MOAA/*S* = 0). During anesthesia maintenance, the infusion rate of remimazolam (0.2–2 mg kg^−1^ h^−1^), and remifentanil (plasma concentration 0.5–2.5 ng ml^−1^) were regulated to ensure that the MOAA/S score reached 0, the BIS maintained between 50 and 65 based on our clinical evidence [27], and the operation was performed without behavioral responses. The MOAA/S was confirmed by the grimace or body movement after sharking the shoulders at 30 s intervals. If signs of intraoperative awakening were detected, the remimazolam dose was adjusted to 10 mg kg^−1^ h^−1^ for up to 1 min. If signs of awakening persisted, remimazolam was discontinued and replaced with propofol. Ephedrine and atropine were used to treat hemodynamic instability.

Hysteroscopy was performed according to clinical practice guidelines. Remimazolam and remifentanil were discontinued at the end of the surgery. All patients were sent to the post-anesthesia care unit (PACU) for recovery.

### Outcome measures

The primary outcome was the time from remimazolam administration to LOC. The secondary outcomes included the doses of remimazolam until LOC, characteristics of the operation and anesthesia, and vital signs. The NIBP, heart rate (HR), SpO_2_, respiratory rate (RR), and BIS were recorded before anesthesia, at LOC, at the starting of the surgery, at the end of the surgery, and at the time of eye opening. The total dose of remimazolam and remifentanil was recorded. The following time parameters were recorded: duration of surgery, duration of anesthesia, and time from drug withdrawal to eye opening. Ramsay scores 15 min after surgery, numerical rating scale (NRS) before leaving the PACU, and crystalloid infusion volume were also recorded. The following adverse events were recorded: hypertension (≥30% increase in mean blood pressure from baseline), hypotension (≤30% decrease in mean blood pressure from baseline), and bradycardia (<50 beats min^−1^), tachycardia (>100 beats/min), and respiratory depression (respiratory rate ≤8 breaths min^−1^ apnea, ≥15 s, SpO_2_ <90% for at least 15 s; or ETCO_2_ ≥50 mmHg), chin lift, cough, injection pain, pharyngeal inlet, dysphoria, nausea or vomiting, awareness, or delirium.

### Statistical analysis

The sample size was calculated using PASS (version 11.0; NCSS, Utah, USA). Our preliminary experiments showed that the time from anesthesia induction to LOC (mean [SD]) was 154 s (27) and 143 s (22) in the saline and remimazolam-1 groups, respectively. Therefore, 80 samples were required for each group (two-sample *t* test power analysis; two-sided alpha = 5%, power = 80%). Considering a dropout rate approximately 10%, the sample size was added to 88 in each group.

Statistical analysis was performed using the SPSS version 26.0 software (IBM, Chicago, IL, USA). Continuous variables with normal distribution (tested by the Shapiro–Wilk test) and equal variances (tested by the Levene test) are expressed as means (standard deviation [SD]). Continuous values with non-normal distribution or unequal variance are expressed as medians (interquartile range [IQR]). Categorical and qualitative variables are reported as frequencies (%). Vital signs such as systolic blood pressure (SBP), diastolic blood pressure (DBP)), mean blood pressure (MBP), heart rate, SpO_2_, respiratory rates, and BIS were compared with repeated-measured two-way analysis of variance (ANOVA), followed by Bonferroni’s multiple comparison. Age, height, weight, and BMI were compared using one-way ANOVA, followed by Bonferroni’s multiple comparison test. Other continuous data were compared using Kruskal–Wallis one-way ANOVA, followed by Dunn’s comparison. Categorical data were analyzed using the Chi-square test (when <20% cells had an expected count less than 5) or Fisher’s test (when ≥20% cells had an expected count less than 5, or at least one cell had an expected count less than 1). Statistical significance was set at a *p* values <0.05 (2-sided).

## Results

### Patients

**Figure 1.**
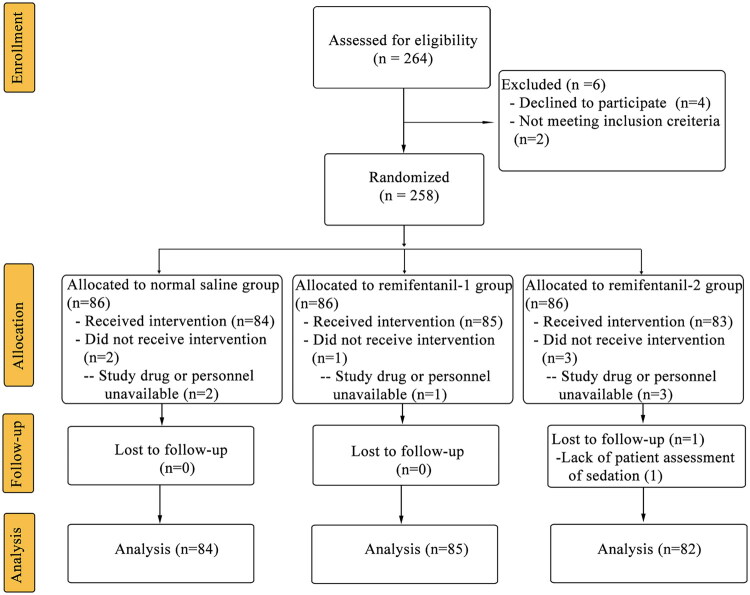
CONSORT flow of clinical procedures. CONSORT indicates consolidated standards for reporting of trials.

A total of 264 patients were recruited between Aug 26, 2022 and Dec 31, 2023. Two hundred and 58 patients were randomly allocated and 251 completed the study (Figure 1). Baseline characteristics of subjects were similar between groups (Table 1).

**Table 1.** Baseline characteristic of patients receiving remimazolam combined with or without remifentanil for anaesthesia induction.

Parameters	Normal saline group (*n* = 84)	Remifentanil-1 group (*n* = 85)	Remifentanil-2 group (*n* = 82)	*p* value
Age; years	37.6 (8.5)	39.2 (9.7)	36.4 (8.3)	0.128
Height; cm	157.9 (5.8)	157.7 (5.6)	157.7 (5.1)	0.974
Body weight; kg	55.5 (8.8)	56.5 (10.9)	53.7 (8.8)	0.160
BMI; kg m^–2^	22.2 (3.2)	22.7 (4.1)	21.5 (2.9)	0.079
Mallampati score				0.235
I	80(95%)	75(88%)	76(93%)	
II	4(5%)	10(12%)	6(7%)	
ASA physical status				0.414
I	79(94%)	75(88%)	74(90%)	
II	5(6%)	10(12%)	8(10%)	

BMI, body mass index; ASA, American Society of Anesthesiologists.

Values are mean (SD) or frequency (%).

### Primary outcome

The primary outcomes are shown in Table 2. The median [IQR] time from anesthesia induction to LOC was significantly shorter in the remimazolam-2 (117.0 [105.9, 135.0] s) and remimazolam-1 (120.0 [104.4, 139.8] s) groups than in the normal saline group (154.5 [139.8, 180.0] s) (*p* < 0.001). No difference was found between the remimazolam-1 and remimazolam-2 groups at the time of the LOC.

**Table 2.** Sedation characteristic of patients receiving remimazolam combined with or without remifentanil for anaesthesia induction.

Parameters	Normal saline group (*n* = 84)	Remifentanil-1 group (*n* = 85)	Remifentanil-2 group (*n* = 82)	*p* value
Time from anaesthesia induction to LOC (s)	154.5 (139.8, 180.0)	120.0 (104.4, 139.8)^#^	117.0 (105.9, 135.0)^#^	<0.001
Total administration of remimazolam from anaesthesia induction to LOC; mg	11.7 (10.4, 13.2)	9.6 (8.7, 10.6)^#^	8.9 (7.9, 10.2)^#,*^	<0.001

Values are presented as median (IQR).

*p = 0.008, compared to the remifentanil-1 group.

^#^*p* < 0.001, compared to the normal saline group.

### Secondly outcomes

The median [IQR] dose of remimazolam from anesthesia induction to LOC was significantly lower in the remimazolam-2 (8.9 [7.9, 10.2] mg) and remimazolam-1 (9.6 [8.7, 10.6] mg) groups than in the normal saline group (11.7 [10.4, 13.2] mg) (*p* < 0.001; Table 2). The median [IQR] dose of remimazolam from anesthesia induction to LOC was significantly lower in the remimazolam-2 group than that in the remimazolam-1 group (*p* = 0.008; Table 2).

Table 3 summarizes the characteristics of anesthesia and surgery. The total dose of remimazolam was significantly lower in the remimazolam-2 (20.7 [17.5, 26.5] mg) and remimazolam-1 (23.2 [20.6, 29.6] mg) groups than in the normal saline group (24.5 [20.6, 29.5] mg) (*p* < 0.001). No significant difference was found in the total dose of remifentanil between the remimazolam-1 and normal saline groups. A significant difference was found in the total dose of remimazolam between the remimazolam-2 and remimazolam-1 groups (*p* < 0.05). The total dose of remifentanil was significantly higher in the remimazolam-2 group (149.3 [108.3, 192.9] mg) than in the remimazolam-1 group (108.0 [90.3, 158.8] mg) and the normal saline group (113.0 [81.5, 152.7] mg) (*p* < 0.001). No significant difference was found in the total dose of remimazolam between the remimazolam-1 and normal saline groups. The time from drug withdrawal to eye opening was shorter in the remimazolam-2 (5.0 [4.0, 7.6] min) and remimazolam-1 (6.0 [4.0, 7.0] min) groups than in the normal saline group (6.7 [5.0, 8.4] min) (*p* = 0.011). There were no differences between the groups in terms of duration of surgery, duration of anesthesia, Ramsay scores at 15 min after surgery, numeric rating scales (NRS) before leaving the PACU, or crystalloid infusion volume.

**Table 3.** Characteristic of anesthesia and surgery in patients receiving remimazolam combined with or without remifentanil for anaesthesia induction.

Parameters	Normal saline group (*n* = 84)	Remifentanil-1 group (*n* = 85)	Remifentanil-2 group (*n* = 82)	*p* value
Total administration of remimazolam; mg	24.5 (20.6, 29.5)	23.2 (20.6, 29.6)	20.7 (17.5, 26.5)^#, *^	<0.001
Total administration of remifentanil; μg	113.0 (81.5, 152.7)	108.0 (90.3, 158.8)	149.3 (108.3, 192.9)^##, ***^	<0.001
Duration of surgery; min	16.2 (11.0, 22.9)	16.4 (13.5, 25.0)	17.0 (11.5, 25.0)	0.462
Duration of anaesthesia; min	26.0 (17.8, 34.4)	27.3 (19.0, 34.0)	26.1 (19.1, 39.4)	0.702
Time from drug withdrawal to opening eyes; min	6.7 (5.0, 8.4)	6.0 (4.0, 7.0)	5.0 (4.0, 7.6)	0.011
Ramsay scores at 15 min after surgery	1.0 (1.0, 1.0)	1.0 (1.0, 1.0)	1.0 (1.0, 1.0)	0.442
NRS before leaving PACU	1.0 (1.0, 1.0)	1.0 (1.0, 1.0)	1.0 (1.0, 1.0)	>0.999
Crystalloid infusion volume; ml	100.0 (100.0, 120.0)	100.0 (100.0, 100.0)	100.0 (100.0, 150.0)	0.408

NRS, numerical rating scale; PACU, post-anesthesia care unit.

Values are presented as median (IQR). ^#^*p* < 0.05, ^##^*p* < 0.01, compared with the normal saline group; *p < 0.05, ***p < 0.001, compared with the Remifentanil-1 group.

**Figure 2.**
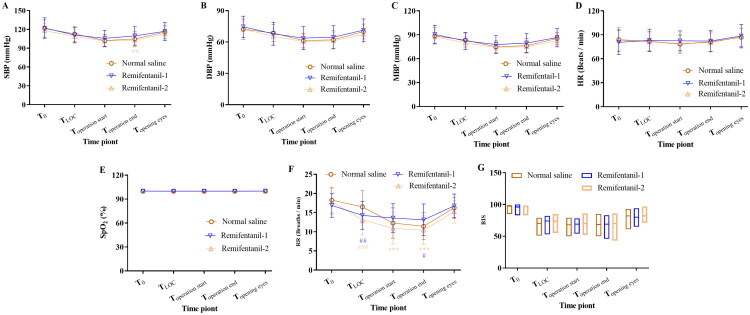
Changes of vital signs during anesthesia. Values are mean (SD) (A–F) or medians (IQR) (G). ^#^*p* < 0.05, ^##^*p* < 0.01, ^###^*p* < 0.001, compared with normal saline group; *p < 0.05, **p < 0.01, ***p < 0.001, compared with remimazolam-1 group. Data were compared using repeated measures two-way analysis of variance (ANOVA) with Geisser–Greenhouse correction, followed by Bonferroni’s multiple comparisons test. A: Drug: *F* (2, 248) = 3.918, *p* = 0.0211; time: *F* (4, 992) = 197.2, *p* < 0.0001; drug × time: *F* (8, 992) = 2.408, *p* = 0.0141; subject: *F* (248, 992) = 7.931, *p* < 0.0001. B: Drug: *F* (2, 248) = 3.885, *p* = 0.0218; time: *F* (4, 992) = 145.9, *p* < 0.0001; drug × time: *F* (8, 992)  = 1.519, *p* = 0.1461; subject: *F* (248, 992) = 6.738, *p* < 0.0001. C: Drug: *F* (2, 248) = 4.414, *p* = 0.0131; time: *F* (4, 992) = 198.5, *p* < 0.0001; drug × time: *F* (8, 992) = 1.793, *p* = 0.0747; subject: *F* (248, 992) = 7.899, *p* < 0.0001. D: Drug: *F* (2, 248) = 0.2131, *p* = 0.8083; time: *F* (4, 992) = 38.85, *p* < 0.0001; drug × time: *F* (8, 992) = 3.228, *p* = 0.0012; subject: *F* (248, 992) = 10.65, *p* < 0.0001. E: Drug: *F* (2, 248) = 0.1519, *p* = 0.8591; time: *F* (4, 992)  = 0.5236, *p* = 0.7184; drug × time: *F* (8, 992) = 2.036, *p* = 0.0396; subject: *F* (248, 992) = 2.530, *p* < 0.0001. F: Drug: *F* (2, 248)  = 8.822, *p* = 0.0002; time: *F* (4, 992) = 192.6, *p* < 0.0001; drug × time: *F* (8, 992) = 10.75, *p* < 0.0001; subject: *F* (248, 992) = 3.808, *p* < 0.0001. G: Drug: *F* (2, 248)  = 1.315, *p* = 0.2704; time: *F* (4, 992)  = 1247, *p* < 0.0001; drug × time: *F* (8, 992)  = 4.641, *p* < 0.0001; subject: *F* (248, 992) = 2.261, *p* < 0.0001. SBP, systolic blood pressure; DBP, diastolic blood pressure; MBP, mean blood pressure; HR, heart rate; SpO_2_, pulse oximetry; RR: respiratory rate; LOC: loss of consciousness; BIS, bispectral index.

There were no differences between the groups in terms of DBP, heart rate, SpO_2_, and BIS at baseline, at LOC, at the beginning of operation, at the end of operation, and at the time of eye opening (Figure 2B,D,E,G). SBP and MBP were lower in the remimazolam-2 group than in the remimazolam-1 group at the end of the operation, but this difference was not clinically significant (Figure 2A,C). There were differences between the groups in terms of RR at LOC (*p* < 0.01, normal saline groups vs remimazolam-1 groups; *p* < 0.001, normal saline group vs remimazolam-2 group), at the beginning of surgery (*p* < 0.001, remimazolam-1 groups vs remimazolam-2 groups), and at the end of operation (*p* < 0.05, normal saline groups vs remimazolam-1 groups; *p* < 0.001, remimazolam-1 groups vs remimazolam-2 groups) (Figure 2F).

Few participants reported adverse events (Table 4). The most commonly reported adverse events were hypotension, respiratory depression, chin lift, cough, pharyngeal inlet pain, and dysphoria. However, no significant differences were found between the groups. None of the patients developed hypertension, tachycardia, injection pain, nausea/vomiting, awareness, or delirium in any of the groups. Two patients in the remimazolam-2 group developed bradycardia. None of the subjects exhibited death, serious adverse events, or discontinuation due to adverse events.

**Table 4.** Incidence of adverse event in patients receiving remimazolam combined with or without remifentanil for anaesthesia induction.

Parameters	Normal saline group (*n* = 84)	Remifentanil-1 group (*n* = 85)	Remifentanil-2 group (*n* = 82)	*p* value
Hypertension	0 (0%)	0 (0%)	0 (0%)	NA
Hypotension	7 (8.3%)	3 (3.5%)	3 (3.7%)	0.335^b^
Bradycardia (<50 beats/min)	0 (0%)	0 (0%)	2 (2.4%)	0.106^b^
Tachycardia (>100 beats/min)	0 (0%)	0 (0%)	0 (0%)	NA
Respiratory depression	7 (8.3%)	3 (3.5%)	7 (8.5%)	0.342^a^
Chin lift	7 (8.3%)	10 (11.8%)	7 (8.5%)	0.697^a^
Cough	1 (1%)	1 (1%)	1 (1%)	>0.999^b^
Injection pain	0 (0%)	0 (0%)	0 (0%)	NA
Pharyngeal inlet	3 (3.6%)	3 (3.5%)	3 (3.7%)	>0.999^b^
Dysphoria	3 (3.6%)	11 (12.9%)	6 (7.3%)	0.078^a^
Nausea/vomiting	0 (0%)	0 (0%)	0 (0%)	NA
Awareness	0 (0%)	0 (0%)	0 (0%)	NA
Delirium	0 (0%)	0 (0%)	0 (0%)	NA

NA, not applicable.

Values are reported as frequency (%).

^a^Obtained from a Chi-square test.

^b^Obtained from a Fisher’s exact test.

## Discussion

The main finding of our study was that the combination of remifentanil and remimazolam shortened the time from anesthesia induction to LOC and reduced the dose of remimazolam in patients undergoing hysteroscopy. To our knowledge, this study is the first to evaluate the synergistic effect of remimazolam-remifentanil anesthesia during anesthesia induction. Our results confirmed our hypothesis that remifentanil enhances the sedative effect of remimazolam during anesthesia induction in patients undergoing hysteroscopy.

Opioids are commonly administered in clinical procedures. Benzodiazepines act synergistically with opioids. For example, midazolam and morphine act additively for sedation [28]. Midazolam acts synergistically with fentanyl to induce anesthesia induction [23]. Both remifentanil and remimazolam are designed for rapid offset by hydrolytic cleavage, and the combination of the two drugs enables precise control of sedation and recovery. Remifentanil-based sedation is superior to dexmedetomidine for hemodynamic stability and patient’s satisfaction [29]. The sedative effects of remimazolam, midazolam, and propofol were synergistically enhanced when they were combined with remifentanil. The mean sedative doses of remimazolam in combination with remifentanil were reduced by 94%, 98%, and 61%, respectively, compared to cynomolgus monkeys without remifentanil [30]. In our study, the time from anesthesia induction to LOC was significantly shorter in the remimazolam-2 and remimazolam-1 groups than that in the normal saline group. At the same time, the doses of remimazolam from anesthesia induction to LOC were significantly lower in the remimazolam-2 and remimazolam-1 groups than in the normal saline group. A significant difference was observed in the doses of remimazolam until LOC between the remimazolam-2 and remimazolam-1 groups. However, the degree of decrease was lower than that observed in cynomolgus monkeys. This may be because of species differences. Our study demonstrated a high synergy between remimazolam and remifentanil.

The co-administration of drugs improved efficacy and exaggerated adverse effects. Life-threatening complications have been reported. For example, mortality increases when sedatives are combined with opioids for pain reduction or prescribed to patients who abuse opioids or alcohol [31,32]. In our study, the most common adverse events were hypotension, respiratory depression, chin lift, cough, pharyngeal inlet, and dysphoria. Hypotension induced by sedative/anaesthesia is common, and this effect may be exacerbated in combination with opioids. Compared to propofol, remimazolam downregulates the incidence of hypotension [19]. However, there were no differences among the three groups in the present study. These results suggest that the combination of remimazolam and remifentanil (plasma concentration ≤2 ng ml^−1^) for general anesthesia have no effect on the hypotension and respiratory adverse events. No patients developed hypertension, tachycardia, injection pain, nausea/vomiting, awareness, or delirium in any of the groups. All of these results suggest that the combination of remimazolam and remifentanil is safe for general anesthesia.

However, our results should be interpreted in the context of these limitations. First, the external validity of the trial was weakened by the inclusion of subjects and the single-center, single-blinded design. This study included only patients who underwent elective hysteroscopy. Therefore, whether our results be applicable to other surgical settings need further study. Meanwhile, there is potential biases due to the single-blinded design. Second, all patients were from a single center in China. Genetic differences in race also require vigilance when interpreting the results. Third, the number of participants in this study was relatively small. Further studies are necessary to increase the sample size to verify these results. Fourth, MOSS/A immediately before the start of remimazolam infusion was not tested, and we could not distinguish between synergism and additivism for these drugs.

## Conclusion

In conclusion, our findings confirm that remifentanil enhances the sedative effect of remimazolam during anesthesia induction in a dose-dependent manner in patients undergoing hysteroscopy.

## Supplementary Material

Supplementary_Materials.doc

## Data Availability

The data generated during this study are available from the corresponding author upon reasonable request.

## Abbreviations

BMI: body mass index
SBP: systolic blood pressure
DBP: diastolic blood pressure
MBP: mean blood pressure
CI: confidence interval
GABAARs: gamma-aminobutyric acid type A receptor
ASA: American Society of Anesthesiologists
PACU: post-anesthesia care unit
SpO_2_: pulse oximetry
BIS: bispectral index
LOC: loss of consciousness
NRS: numerical rating scale
PONV: postoperative nausea/vomiting
SD: standard deviation
IQR: interquartile range
ASD: absolute standardized difference
ANOVA: analysis of variance
